# Modulation of the Disturbed Motor Network in Dystonia by Multisession Suppression of Premotor Cortex

**DOI:** 10.1371/journal.pone.0047574

**Published:** 2012-10-10

**Authors:** Ying-Zu Huang, Chin-Song Lu, John C. Rothwell, Chung-Chuan Lo, Wen-Li Chuang, Yi-Hsin Weng, Szu-Chia Lai, Rou-Shayn Chen

**Affiliations:** 1 Department of Neurology, Chang Gung Memorial Hospital and Chang Gung University College of Medicine, Taipei, Taiwan; 2 Sobell Department of Motor Neuroscience and Movement Disorders, Institute of Neurology, University College London, Queen Square, London, United Kingdom; 3 Institute of Systems Neuroscience, National Tsing Hua University, Hsinchu, Taiwan; University Medical Center Groningen UMCG, The Netherlands

## Abstract

Daily sessions of therapeutic transcranial brain stimulation are thought to prolong or amplify the effect of a single intervention. Here we show in patients with focal hand dystonia that additional, new effects build up progressively over time, making it difficult to predict the effect of long term interventions from shorter treatment sessions. In a sham-controlled study, real or sham continuous theta burst stimulation (cTBS) was given once daily for five consecutive days to dorsolateral premotor cortex (PMd). Five days of real, but not sham, premotor cTBS improved intracortical inhibition in primary motor cortex (M1) to a similar extent on day 1 and day 5. However 5 days of cTBS were required to restore the abnormal PMd-M1 interactions observed on day 1. Similarly, excessive M1 plasticity seen at baseline was also significantly reduced by five days of real premotor cTBS. There was only a marginal benefit on writing. The results show that additional, new effects, at sites distant from the point of stimulation, build up progressively over time, making it difficult to predict the effect of long term interventions from shorter treatment sessions. The results indicate that it may take many days of therapeutic intervention to rebalance activity in a complex network.

## Introduction

Transcranial brain stimulation is used increasingly as a potential therapeutic intervention for a variety of conditions. Because studies have shown that the after-effects of stimulation can be prolonged when repeated sessions are given [1]–[4], therapy is almost always designed around daily stimulation delivered for weeks or more. The effects of a single session of stimulation are often assumed as valid predictors of long-term changes that might be expected in a therapeutic protocol [5]. However, there is some evidence that the effects of long-term treatment may differ in quality from those of a single session. In depression, effects of repetitive transcranial magnetic stimulation (rTMS) in excess of those of placebo can only be observed after several weeks of treatment. Similarly, a progressively developing response to therapeutic brain stimulation can be observed after implantation of deep brain stimulation (DBS). Maximum clinical effects of DBS in dystonia, as well as Tourette syndrome and obsessive compulsive disorder, may take months to develop [6]–[8]. Indeed, in dystonia, implantation of DBS may initiate progressive changes in underlying motor physiology that are not apparent when testing acutely [9].

The present experiments examined whether repeated sessions of rTMS can promote slow reorganisation in the motor system of patients with writer's cramp (WC). This is a clinically relevant condition in which rTMS to the dorsal premotor cortex (PMd) has already been tested as a potential therapeutic intervention. Although small effects of a single session of have been reported [10], repetitive sessions over consecutive days are usually required for clearer therapeutic effects [11]–[14]. We ask whether repeated sessions of rTMS lead to cumulative effects on typical pathophysiological hallmarks of dystonia that cannot be observed after a single intervention.

Our intervention targeted PMd since functional imaging studies have often revealed that it is hyperactive during movement in patients with dystonia [15]–[18]. However the underlying mechanism of hyperactivity in PMd and its role in causing dystonia remain unclear. It could be an intrinsic premotor deficit or reflect abnormal interaction in a wider motor network. We therefore assessed effects of multisession premotor suppression on both premotor and motor cortex to gain some insight into possible motor network reorganisation in dystonia.

We applied cTBS to PMd for 5 days and measured effects on physiological markers of dystonia: the network interaction from PMd-M1, and the increased plasticity and reduced inhibition within M1 in WC patients [10], [19]–[21]. cTBS is generally believed to suppress the stimulated cortex [22], [23], although recent reports using protocols slightly different from that used in the current study suggest that the response to TBS protocols is variable and the effect of cTBS may not be always inhibitory [24]–[26]. We hypothesised that PMd suppression might restore PMd-M1 connectivity. This would not only be evident as a normalisation in M1 intracortical inhibition as we have observed previously in a single session cTBS study [10], but also might reduce overactive M1 plasticity that is so common in dystonia.

## Materials and Methods

### Ethics Statement

The experiments were performed with the approval of the Institutional Review Board of the Chang Gung Memorial Hospital. All participants gave their informed consent prior to participation.

### Subjects

Eighteen WC patients affecting the dominant right arm and hand (10 men, 42.1±9.8 years) (Table 1) and eight age-matched healthy subjects (3 men, 41.9±9.9 years) were recruited with informed consent and the approval of the Institutional Review Board of the Chang Gung Memorial Hospital in Taiwan. Experiments on patients were performed following 24-hour drug withdrawal.

**Table 1:** Demographic data of patients with focal hand dystonia.

No.	Age	Sex	Onset*	Clinical features	Medication
**REAL**					
**R-1**	49	F	40	Flexion of the right thumb, and index finger, ulnar deviation of the wrist, extension of the elbow	Tri, Clo, Oxa, Bez, BTX
**R-2**	40	M	32	Tightly fist the pen when writing, wrist radial extension, supination of the elbow	Tri, Clo
**R-3**	36	F	24	Flexion of the fingers, extension of the thumb, extension of the wrist, elevation of the shoulder	Bac, Tri, Clo, Top, BTX
**R-4**	32	F	21	Flexion of the thumb, index and middle fingers, extension of the wrist, tremulous writing#	Tri, Clo, Tri, Pro, BTX
**R-5**	34	M	11	Right upper limb bradykinesia and rigid, flexion of the fingers and wrist, tremulous writing#	Tri, Clo, Pro
**R-6**	57	M	37	Tightly holding the pen, flexion of the fingers and wrist, abduction of the elbow	Tri, Clo, Cbz, Bez, BTX
**R-7**	60	F	43	Difficulty in initiation of writing, flexion of fingers, tightly fist the pen, tremulous writing#	Clo, Cba, BTX
**R-8**	37	M	31	Tightly holding the pen, flexion of the fingers and wrist, elevation of the shoulder	Tri, Bez, Bac, Clo, BTX
**R-9**	38	M	25	Tightly holding the pen, nib darting, mild flexion of the wrist, tremulous writing#	Tri, Clo, Cbz, BTX
**SHAM**					
**S-1**	42	F	34	Flexion of the index, 4^th^, 5^th^ fingers, adduction of the thumb, extension of the elbow	Tri, Clo, Bez, Oxa, BTX
**S-2**	35	M	30	Tightly holding the pen, flexion of the fingers, pain over the peri-elbow, tremulous writing#	Tri, Clo, Cbz
**S-3** (x)	38	M	27	Difficulty in initiation of writing, flexion of the index, 3th and 4^th^ fingers, tremulous writing#	Tri, Clo, Oxa
**S-4**	37	F	29	Flexion-extension tremor of the hand when outstretching and acting, wrist abduction and pronation	Tri, Clo, Oxa, Bac
**S-5**	65	M	53	Tightly holding the pen, difficulty in initiation of writing, tremulous writing#	Pro, Clo, Top
**S-6**	35	M	21	Initially presenting right hand clumsy with flexion posture in writing, then difficulty in playing flute	Tri, Clo, Oxa, Top
**S-7**	47	F	40	Flexion of thumb and index fingers, radial extension of the wrist and elbow	Tri, Clo
**S-8** (o)	33	M	27	Tightly holding the pain with flexed fingers, flexion of the wrist, tremulous writing#	Tri, Clo, Tri, Pro, BTX
**S-9** (o)	43	F	38	Clumsy with fingers flexion on writing, elevation of the shoulder.	Tri, Clo, Oxa

No.: the anonymised patient identification numbers in each of the two groups; *: the age at onset of years; #: denote the abnormal posture when writing showed jerky and tremulous dystonic movement; (x): dropped out; (o): hand writing tests only.

Oxa: oxcarbazepine; Tri: trihexyphenidyl; Clo: clonazepam; Top: topiramate; Cbz: carbamazepine; Pro: propranolol; Bez: benzodiazepam; Bac: baclofen.

BTX (botulinum toxin A injection): the timing of the last injection before the experiment is 6 month in R-8 and S-1 and >12 months in R-1, R-3, R-4, R-6, R7, R-9 and S-8.

### Experimental Design

#### Main experiment (Fig 1A)

**Figure 1:**
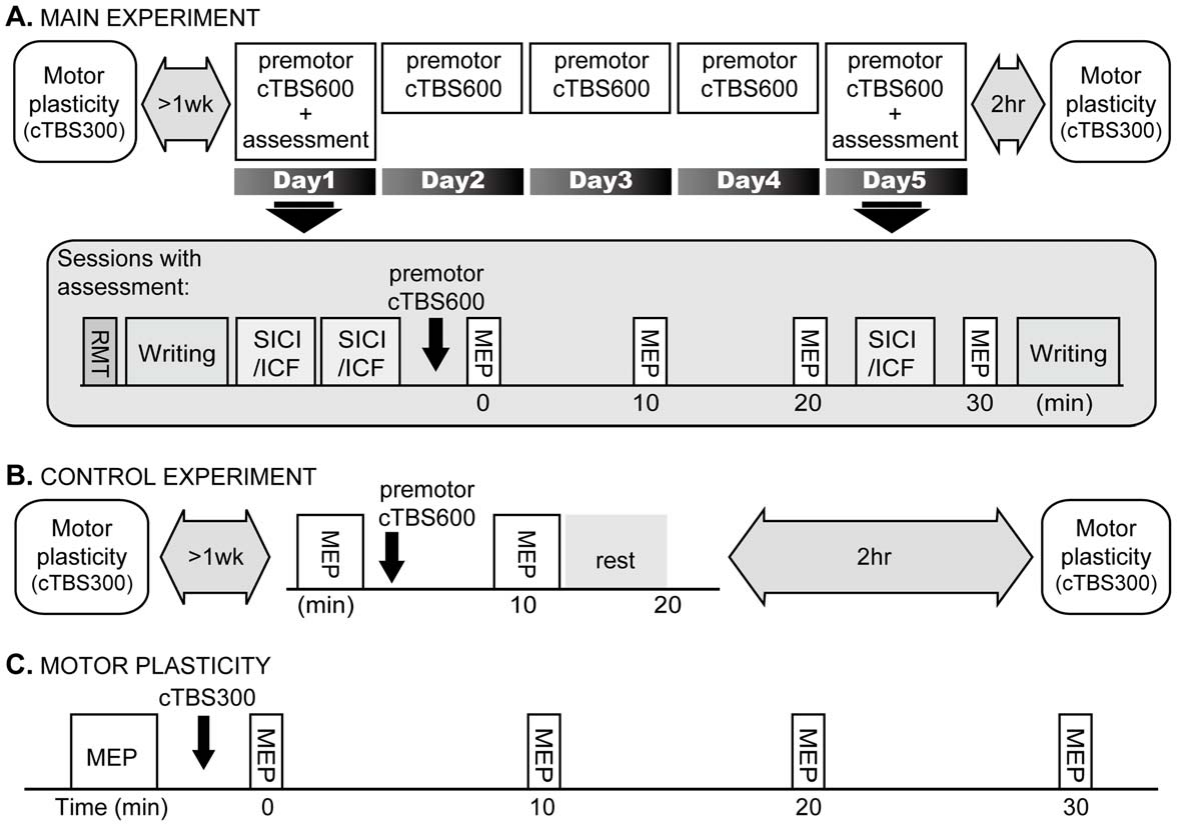
Experimental design. In the main experiment (A), dystonic patients received premotor cTBS600 on five consecutive days. Rest motor threshold (RMT) was assessed at the beginning of the experiment on day 1 and day 5. The amplitude of MEPs, writing tests and SICI/ICF were recorded before and after premotor cTBS600 on day 1 and 5. Motor plasticity assessed by cTBS300 given to M1 was measured more than one week before or one month after the 5-day premotor cTBS600 and 2 hours after premotor cTBS600 on day 5. In a control study (B), only a single session of premotor cTBS600 was given to dystonic and healthy subjects. The amplitude of MEPs was recorded before and after premotor cTBS600. Motor plasticity was assessed more than one week before or one month after premotor cTBS600 and 2 hours after premotor cTBS600. Motor plasticity was assessed by the change in the size of MEP that is induced by cTBS300 given to M1 (C).

Patients were randomly assigned into real (9 patients: 5 men, 42.6±10.2 years) and sham (9 patients: 5 men, 41.7±9.8 years) groups. In the sham group, one patient dropped out due to personal reasons and two patients had only hand writing assessed. Patients came for 5 consecutive days to have real or sham cTBS for 40 s (cTBS600) over left PMd (premotor cTBS600). On day 1 and 5, rest motor threshold (RMT), hand writing assessed with writing speed and Gibson Spiral Maze tests and two blocks of short-interval intracortical inhibition (SICI) and intracortical facilitation (ICF) were recorded before cTBS600. The unconditioned test motor evoked potentials (MEPs) recorded in the two blocks of SICI/ICF were considered as baseline MEPs. After premotor cTBS600, MEPs were assessed at 0, 10, 20 and 30 min after cTBS600. Between 20 and 30 min after cTBS600, one block of SICI/ICF was recorded. After the last block of MEP recording, hand writing was reassessed. After the assessments on day 5, patients reported subjective improvement in writing on a 6-point scale, then left the laboratory and returned 2 hours later for motor plasticity assessment (Fig. 1C).

#### Control experiment (Fig 1B)

In the main experiment motor plasticity was tested 2 hrs after the end of the last treatment session on day 5. The control experiment tested whether effects required 5 days of premotor stimulation or whether they also occur 2 hrs after a single treatment session. Eight of the dystonic patients (5 from the real group) and all healthy subjects participated. Twenty baseline MEPs assessed every 4.5–5.5 seconds were recorded. Then premotor cTBS600 was given followed by 20 MEPs at 10 min after cTBS600. After 10 min rest, subjects left the laboratory and then returned 2 hours later to have motor plasticity assessed. The control experiment was performed at least one month apart from the main experiment.

### Data Recording

Subjects were seated in a comfortable chair. EMGs were recorded from the right FDI. Signals were sampled at 5 kHz, amplified with a gain of 1000 and 5000 and filtered (3 Hz to 2 kHz). The surface EMG of the FDI was continuously monitored by an oscilloscope throughout the experiments. Trials in which the target muscle was not relaxed were rejected online.

Single- and paired-pulse TMS was given using a 70 mm figure-of-eight coil connected to a Magstim BiStim^2^ (Magstim Co., UK), whereas TBS was produced by a Magstim Rapid^2^ Package through another 70 mm figure-of-eight coil. The coil was placed over the left hemisphere tangentially to the scalp with the handle pointing backwards. The “motor hot-spot” was defined as the location where TMS produced the largest MEP from FDI. RMT was defined as the minimum stimulation intensity over the hot-spot that could elicit an MEP of no less than 50 uV in five out of ten trials. Active motor threshold (AMT) was defined as the minimum stimulation intensity over the “motor hot-spot” that could elicit an MEP of greater than 200 µV in five out of ten trials during voluntary contraction of FDI. MEP assessment was assessed with single-pulse TMS in trains of 12 pulses (unless specified) given every 4.5–5.5 seconds, and the intensity was set to that required to produce an MEP of approximately 1 mV in the baseline condition and remained unchanged throughout the experiment.

#### Theta burst stimulation (TBS)

Two TBS protocols were used in the present study: cTBS600 and cTBS300 that contains 3-pulse 50 Hz bursts at 80% AMT given every 200 ms for 40 s and for 20 s, respectively [22], [27], [28]. cTBS600 was given over left PMd, which was located as being 2.5 cm anterior to the “motor hot-spot” [29], [30], while cTBS300 was given over the left M1.

In the sham stimulation, the coil was flipped over to stimulate with the flip side and the stimulus intensity was reduced to 60% of AMT. We have compared RMT measured with the normal side and flip side on 20 healthy subjects. The mean RMT ± S.D. measured with the normal side was 45.3±10.6% of maximum stimulator output (MSO), while that measured with the flip side was 58.1±14.0% of MSO. In other words, the output of the flip side is about 78% of the normal side. Hence, we stimulated the sham group at a much lower intensity (approximately 46.8% AMT), but the stimulation was felt and sounded very similar to the real stimulation. We have demonstrated that cTBS at around 60% AMT given to PMd produces no effect on MEPs [30].

#### Motor cortex plasticity in response to cTBS300 over M1

Motor plasticity was evaluated using cTBS300. AMT was assessed during a tonic voluntary contraction for 3 min starting 5 min before baseline MEPs were measured using 30 pulses delivered every 4.5–5.5 seconds. cTBS300 was then applied to M1. Following this, MEP size was assessed using 12 pulses given every 4.5–5.5 seconds at 0, 10, 20 and 30 min after the end of cTBS300. Baseline motor plasticity was measured more than one week before or one month after the main and control experiments.

#### SICI/ICF

SICI/ICF was assessed using a paired-pulse technique [31] with the conditioning stimulus at 80% AMT and the test stimulus at an intensity producing an MEP of 1 mV. Subjects received in a random order either the test stimulus alone (test MEPs), or conditioning-test stimuli (conditioned MEPs) at interstimulus intervals (ISIs) of 3, 7 and10 ms for a total of eight trials per condition. The inter-trial interval was 4.5–5.5 s. If necessary, we adjusted the test stimulus intensity while assessing SICI/ICF after premotor cTBS600 to maintain the amplitude of test MEPs at approximately 1 mV.

#### Hand writing tests

Although this is not the major goal of the present study, we assessed the functional effect on hand writing. Writing speed and Gibson Spiral Maze test [32], [33] were tested with pen and paper, and were videoed for off-line analysis. For the writing speed test, subjects copied a page of Chinese as quickly as possible in 3 minutes and the number of characters copied was counted. In the Spiral Maze test, subjects traced the path in the maze from the centre outward with a pen. Errors were scored as the frequency with which the tracing touched any obstacles or the maze border. Subjects were instructed to trace as quickly as possible and avoid errors if possible. All subjects practiced twice before assessment. In addition, after 5 day stimulation, subjective improvement in writing was reported on a 6-point scale as follows: 0 no improvement, 1 minimal improved, 2 mildly improved, 3 moderate improved, significantly improved, 5 fully recovered.

### Data Analysis

Data were analyzed using SPSS. For the effects on PMd-M1 connectivity and motor plasticity, a three-way repeated measures ANOVA was performed to compare the results before and after premotor intervention of the patients with real and sham stimulation or healthy controls. A two-way followed by one-way ANOVA was used to examine the time course of changes in MEP in individual groups. The averaged peak-to-peak amplitudes of MEP at each time block were used for analysis. For the results of SICI/ICF and writing tests, a two-way ANOVA was used to examine the changes between groups, and a one-way ANOVA was used to examine the course within each group. SICI and ICF were calculated as the ratio of the mean conditioned and test MEPs. Post-hoc paired t-tests were used to compare between time points if needed. A P<0.05 was considered statistically significant.

## Results

### Effect of Daily Premotor cTBS on PMd-M1 Interaction

**Figure 2:**
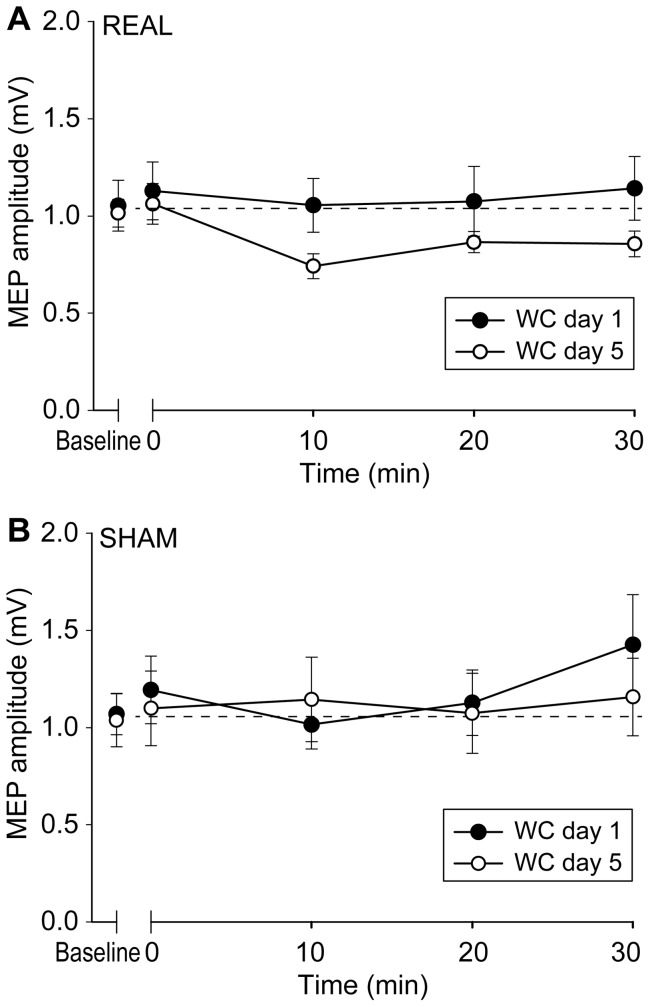
PMd-M1 connectivity in dystonia. In the group that had real stimulation (A), premotor cTBS600 did not change M1 excitability on day 1, while the usual suppression of excitability was restored on day 5. In the group that had sham stimulation (B), no effect was found on MEPs on either day 1 or day 5. Error bars refer to the standard error of the measurements (SEM).

RMT on day1 and day 5 were not significantly different in both real (p = 0.480) and sham (p = 0.264) groups. The amplitudes of baseline MEPs were equal in the real (day 1: 1.05±0.39 mV; day 5: 1.03±0.21 mV) and sham groups (day 1: 1.20±0.26 mV; day 5: 1.11±0.36 mV). This was confirmed by a two way ANOVA showing no effect of GROUP (real and sham) (p = 0.372) and DAY (day 1 and 5) (p = 0.615), and no GROUP × DAY interaction (p = 0.791). We then compared the effect of premotor cTBS600 on MEP amplitudes on the first and fifth days in real and sham groups using three-way ANOVA with a between-subject effect of GROUP (real and sham) and within-subject effects of DAY (day 1 and 5) and TIME (before, 0, 10, 20 and 30 min after premotor cTBS600). There was a significant GROUP × DAY × TIME interaction (F(4,52) = 3.043, p = 0.025) and a significant effect of TIME (F(4, 52) = 2.939, p = 0.029). A further two-way ANOVA showed a significant DAY × TIME interaction (F(4, 32) = 3.775, p = 0.013) in the patients with real stimulation (Fig. 2A). This was because premotor cTBS600 had no effect on MEPs on day 1 (F(4, 32) = 0.433, p = 0.784), but suppressed MEPs on day 5 (F(4, 32) = 8.028, p<0.001). In contrast, sham cTBS600 had no effect of DAY or TIME and no DAY × TIME interaction (p = 0.827, 0.157 and 0.330, respectively), suggesting that sham stimulation produced no effect on MEPs on either day 1 or day 5 (Fig. 2B).

### Effect of Daily Premotor cTBS on SICI/CIF

**Figure 3:**
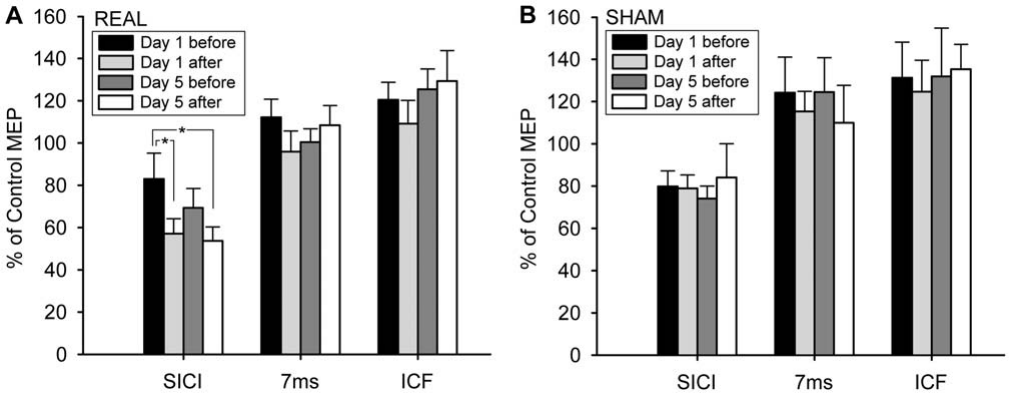
The effect of premotor cTBS600 on SICI/ICF. In the real group (A), SICI was enhanced by premotor cTBS600, while ICF and the paired-pulse excitability at ISI of 7 ms remained unchanged. In the sham group (B), SICI, ICF and the paired-pulse excitability at ISI of 7 ms were not changed. Error bars refer to SEM.

A two-way ANOVA comparing SICI at an ISI of 3 ms between real and sham groups showed a significant GROUP × TIME (day 1 before, day 1 after, day 5 before and day 5 after) interaction (F(3,39) = 2.881, p = 0.048) (Fig. 3). A further one-way ANOVA confirmed that was because SICI changed with TIME (F(3,24) = 5.613, p = 0.005) in the real group, while SICI did not change in the sham group (F(3,15) = 0.326, p = 0.807). Post-hoc analysis revealed that SICI was significantly enhanced on day 1 (p = 0.038) and tend to be enhanced on day 5 (p = 0.074) after premotor cTBS600 as compared to the baseline SICI on Day 1. None of the other pair-wise comparisons of SICI between days and within a day was significant. As regards the result at ISI  = 7 ms and ICF at ISI  = 10 ms, there was no significant effect of GROUP (p = 0.311 and 0.492, respectively), TIME (p = 0.366; and 0.536, respectively) or GROUP × TIME interaction (p = 0.509 and 0.976, respectively) between real and sham groups, indicating that neither real nor sham premotor cTBS changed these two parameters.

### Effect of Daily Premotor cTBS on Hand Writing Tests

**Figure 4:**
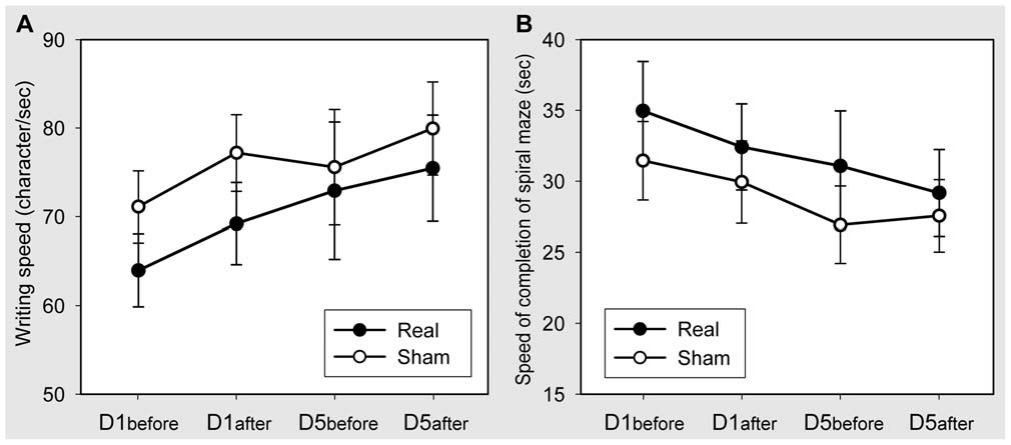
The effect of premotor cTBS600 on hand writing tests. The writing speed was increased by premotor cTBS600 in both the real and sham groups (A). Similarly, both the real and sham groups completed the spiral maze test faster after premotor cTBS600 (B). Error bars refer to SEM.

All patients reported subjective improvement in writing after 5 day stimulation, while the real group (average score: 2.89±0.93; range: 1–4) improved more than the sham group (average score: 1.75±0.71; range: 1–3) (t = 2.816, p = 0.012). We compared the writing speed between real and sham groups using a two-way ANOVA. There was an effect of TIME (F(3,45) = 6.055, p = 0.001), but no effect of GROUP (F(1,15) = 0.746, p = 0.401) or GROUP × TIME interaction (F(3.45) = 1.150, p = 0.339). This was because the writing speed increased significantly in the real group (F(3,24) = 4.145, p = 0.017) and marginally significantly in the sham group (F(3,21) = 3.057, p = 0.051) (Fig. 4A). Similarly, a comparison of speed of completion of the spiral maze showed an effect of TIME (F(3,45) = 7.660, p<0.001), but no effect of GROUP (F(1,15) = 0.487, p = 0.496) or GROUP × TIME interaction (F(3,45) = 0.493, p = 0.689). Both groups completed the spiral maze test faster after premotor cTBS600 (real: F(3,24) = 3.403, p = 0.034; sham: F(3,21) = 6.930, p = 0.002) (Fig. 4B). There was no TIME or GROUP effect and no GROUP × TIME interaction between the two groups in the number of error occurring during the spiral maze test.

### Effect of Daily Premotor cTBS on Motor Cortex Plasticity

**Figure 5:**
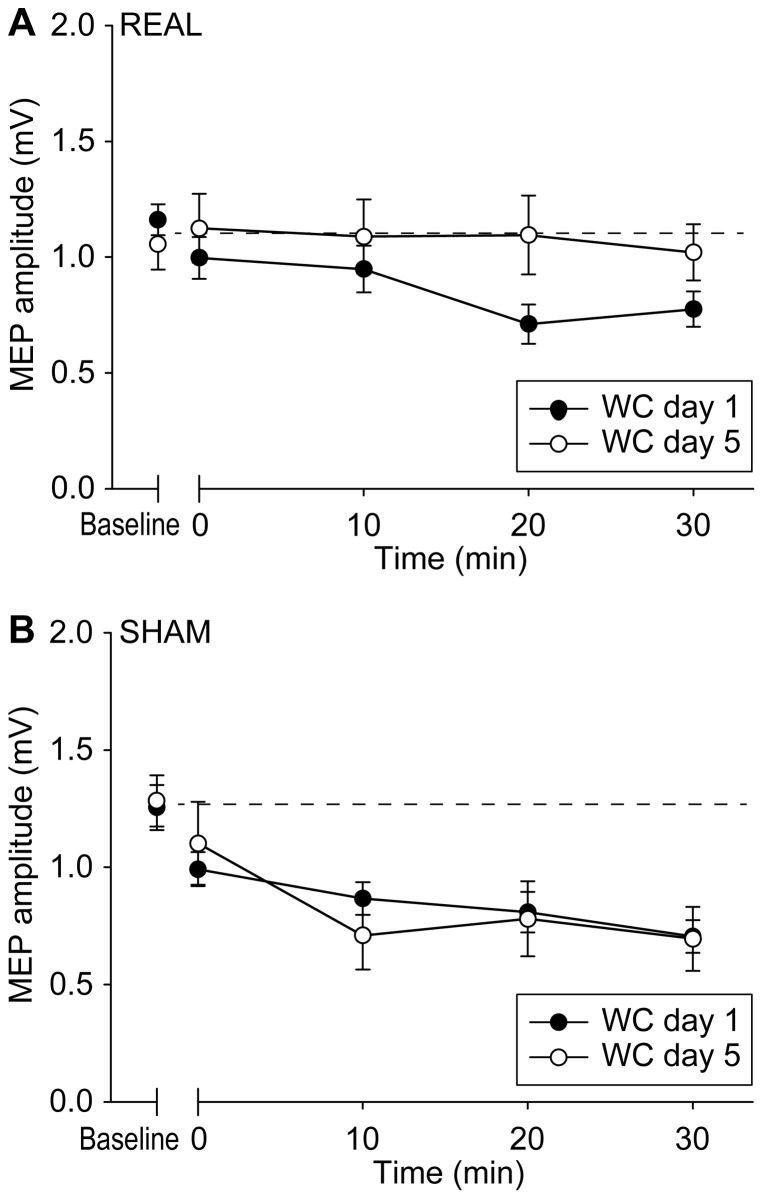
The effect of premotor cTBS600 for five consecutive days on motor plasticity in dystonia. The motor plasticity-like effect induced by cTBS300 given to M1 was significantly reduced or abolished after 5 days of real premotor stimulation (A), while motor plasticity remained unchanged after sham premotor stimulation (B). Error bars refer to SEM.

The amplitudes of baseline MEPs were not different between real (baseline: 1.20±0.20 mV; day 5: 1.06±0.33 mV) and sham groups (baseline: 1.26±0.23 mV; day 5: 1.28±0.13 mV). This was confirmed by a two way ANOVA showing no effect of GROUP (real and sham) (p = 0.200) and DAY (baseline and day 5) (p = 0.544), and no GROUP × DAY interaction (p = 0.369). In order to analyse the effect of daily premotor cTBS on motor cortex plasticity we conducted a three-way ANOVA with the effects of GROUP (real and sham), DAY (baseline and day 5) and TIME (before, 0, 10, 20 and 30 min after premotor cTBS600). This showed significant GROUP × TIME × DAY (F(4, 52) = 2.584, p = 0.048) and GROUP × TIME (F(4, 52) = 4.646, p = 0.003) interactions and a significant TIME effect (F(4, 52) = 16.886, p<0.001). In the group with real stimulation, a two-way ANOVA showed a significant DAY × TIME interaction (F(4,.32) = 4.077, p = 0.009) (Fig. 5A). This was because cTBS300 to M1 significantly suppressed MEPs in the baseline condition (F(4, 32) = 7.971, p<0.001), while the effect of cTBS300 on MEPs disappeared after 5-days of premotor stimulation (F(4, 32) = 0.127, p = 0.972). On the contrary, there was a significant TIME effect (F(4, 20) = 13.564, p<0.001), but no DAY effect (p = 0.941) or DAY × TIME interaction (p = 0.510) in the sham group, suggesting that sham stimulation did not modify the suppression effect of cTBS300 (Fig. 5B). Moreover, a two-way ANOVA on the baseline motor cortex plasticity showed a significant GROUP × TIME interaction between all dystonic patients and healthy subjects in the control experiment (F(4,84) = 2.996, p = 0.023), suggesting that at baseline, cTBS300 over M1 produced an excessive motor plasticity-like effect in patients as reported previously [10], [20].

### Control Experiment

We first evaluated the effect of premotor cTBS600 on the size of MEPs in patients and controls. A two-way ANOVA with factors of TIME (before, 10 min after) and GROUP (patient, control) showed a significant TIME × GROUP interaction (F(1,14) = 4.671, p = 0.048). This was because premotor cTBS suppressed MEPs from 1.16±0.26 mV to 0.82±0.26 mV in healthy controls (t = 3.520, p = 0.010), but not in dystonic patients (baseline: 1.28±0.35 mV, 10 min after: 1.29±0.64 mV; t = −0.1747, p = 0.887). In addition, the amplitude of MEPs measured at 2 hrs after premotor cTBS600 and before cTBS300 to M1 had returned to baseline in both control and patient groups (p = 0.783 and 0.956, respectively).

The amplitudes of baseline MEPs in the motor cortex plasticity sessions were not different between patients (baseline, >1 week prior to premotor cTBS: 1.27±0.38 mV; 2 hours after premotor cTBS: 1.34±0.47 mV) and controls (baseline, >1 week prior to premotor cTBS: 1.19±0.23 mV; 2 hours after premotor cTBS: 1.09±0.20 mV). This was confirmed by a two way ANOVA showing no effect of GROUP (patients and controls) (p = 0.227) and DAY (baseline and 2 hours after premotor cTBS) (p = 0.886), and no GROUP × DAY interaction (p = 0.391). We next compared whether the response to motor cortex cTBS300 was the same at baseline as when tested 2 hrs after premotor cTBS600. A three-way ANOVA with the effects of GROUP (patient and control), DAY (baseline and 2 hours after premotor cTBS) and TIME (before, 0, 10, 20 and 30 min after premotor cTBS600) showed significant DAY × GROUP × TIME (F(4, 56) = 2.645, p = 0.043) and GROUP × TIME (F(4, 56) = 5.422, p = 0.001) interactions and significant DAY (F(1, 14) = 6.149, p = 0.026) and TIME (F(4, 56) = 5.183, p = 0.001) effects.

**Figure 6:**
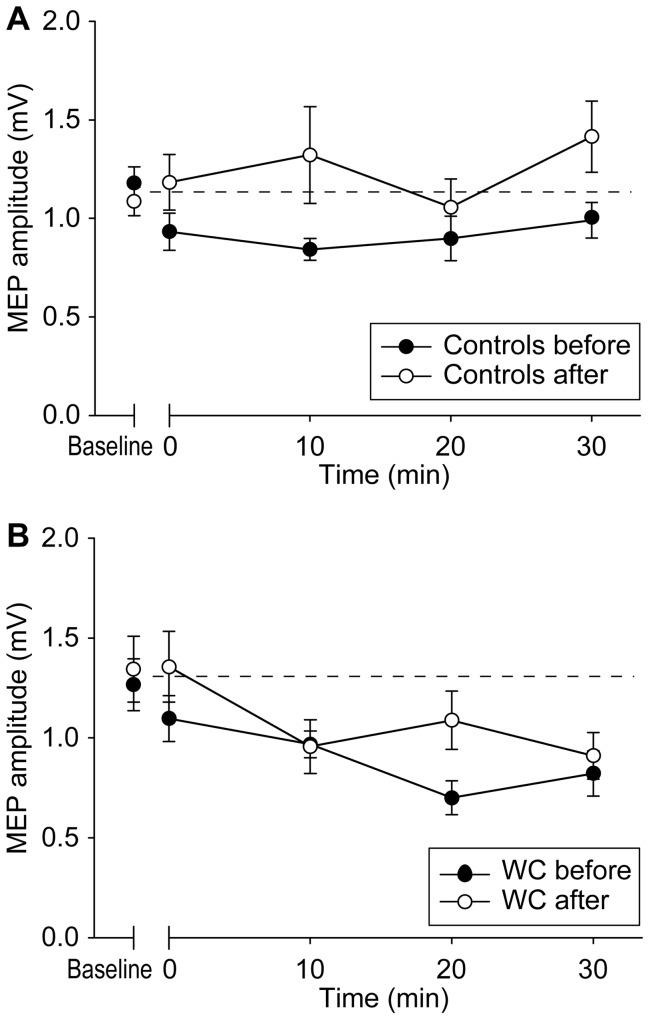
The effect of a single session of premotor cTBS600 on motor plasticity. A single session of premotor cTBS600 significantly reduced the motor plasticity produced by cTBS300 to M1 in healthy subjects (A). In contrast, a single session of premotor cTBS600 only produced marginal effect on motor plasticity in dystonic patients (B). Error bars refer to SEM.

We next investigated the effect of TIME. In the control group, a two-way ANOVA showed a significant DAY × TIME interaction (F(4,28) = 3.204, p = 0.028) (Fig. 6A). In the baseline condition, there was a significant TIME effect (F(4,28) = 3.337, p = 0.024) suggesting that MEPs were successfully suppressed by cTBS300 over M1. In contrast, cTBS600 given 2 hours before to premotor cortex blocked the effect of motor cortex cTBS300 (F(4,28) = 1.964, p = 0.128). In the patient group, a two-way ANOVA showed neither DAY effect (F(1,7) = 2.129, p = 0.188) nor DAY × TIME interaction (F(4,28) = 1.444, p = 0.246) (Fig. 6B). cTBS300 significantly suppressed MEPs in both conditions (baseline: F(4, 28) = 6.608, p = 0.001; 2 hours after premotor cTBS: F(4,28) = 3.517, p = 0.019). Thus, the data appeared to suggest that in healthy individuals, a single session of premotor cTB600 could abolish the plasticity-like effect of motor cortex cTBS, even when given2 hrs later, whereas this effect was much smaller or absent in patients. Subsequent two way analyses revealed a significant GROUP × TIME interaction between patients and controls in the baseline motor plasticity (F(4,56) = 2.823, p = 0.033), confirming that cTBS300 over M1 produced a more profound longer-lasting motor plasticity-like effect on patients.

## Discussion

The present data show that daily sessions of rTMS can have cumulative effects on motor system physiology that are not evident after a single period of stimulation. On the first day of premotor stimulation, cTBS600 over PMd enhanced SICI, but failed to suppress MEPs in dystonic patients. After five consecutive days of premotor stimulation, cTBS600 over PMd successfully reduced the size of MEPs, although the amount of SICI was not further enhanced. In addition, the excessive plasticity-like effect induced by cTBS300 over M1 disappeared after five days of real, but not sham, premotor stimulation. Patients who had real stimulation reported a better subjective improvement than those had sham stimulation. However, this was not evident in the objective measures of writing performance: both real and sham premotor stimulation improved in writing speed and spiral maze tests.

### PMd-M1 Interaction

The lack of effect of premotor cTBS600 on M1 excitability on the first day of stimulation confirms that the PMd-M1 interaction is reduced in dystonia [10], [21], [34]. The new data shows that the suppressive effect of premotor cTBS on M1 excitability can be restored to some extent after five daily sessions of premotor cTBS600. Many imaging studies have reported PMd hyperactivity in dystonia, and this is possibly compensated by reduced PMd-M1 interactions. If 5 days of premotor cTBS600 reduced overactivity of PMd this would reverse the process and normalise PMd-M1 interaction. An alternative possibility, that PMd is hyperactive because of reduced PMd-M1 interaction seems less likely, since suppression of PMd could occur without affecting PMd-M1 interaction.

### SICI/ICF

As reported previously [10], premotor cTBS600 enhanced abnormal SICI towards normal levels in WC patients even on day 1 stimulation. However, there was no further improvement in SICI after five-days of PMd stimulation: after premotor cTBS the amounts of SICI are very close to the values in healthy subjects tested with the same protocol [30]. Hence, it is likely that a single session of premotor cTBS600 has reached the maximum effect that premotor cTBS600 can have on SICI in dystonia, and multisession stimulation brings no further improvement. There was no difference in the amount of SICI immediately before premotor cTBS on day 5 compared with baseline SICI on day1, indicating that repeated sessions of premotor cTBS do not produce longer-lasting (>24 hrs) effects on SICI. However, since drugs were withdrawn 24 hours before the first cTBS600 session, we cannot exclude the possibility that the longer period of benzodiazepine withdrawal on Day 5 counteracts the effect of multisession premotor stimulation on GABAergic SICI.

### Motor Cortex Plasticity

In the baseline condition, cTBS300 over M1 produced an excessive plasticity-like response in patients as reported previously with a variety of protocols [19], [20]. Interestingly, cTBS300 over M1 no longer produced an after-effect on MEP size after five days of premotor stimulation. This combination of restored PMd-M1 interaction together with reduced M1 plasticity mimics the pattern that we had previously observed in non-clinically manifesting DYT1 mutation carriers [10], [20]. A reduced M1 plasticity responding to paired associative stimulation is also seen in dystonic patients after DBS [35], [36]. Hence modification of motor cortex plasticity could be one of the underlying mechanisms of the therapeutic benefit caused by rTMS over the premotor area [11]–[14].

Interpretation of the mechanism of the effect of 5 days' premotor cTBS600 on motor plasticity was, however, slightly complex. Our control experiment showed that a single session of premotor cTBS600 in healthy individuals abolished their response to the motor plasticity protocol even when it was tested 2 hrs later. In contrast, the effect of a single session was minimal in patients; 5 consecutive sessions of premotor cTBS600 were required before it abolished motor cortex plasticity as in healthy subjects. We suggest that the smaller effect seen in dystonic patients on day 1 was due to reduced PMd-M1 connectivity at that time. Five days of premotor stimulation restored the connectivity and therefore restored the modulatory effect of premotor cTBS600 on motor plasticity. However, we cannot rule out the possibility that an overactive premotor cortex requires repetitive suppression to show consequent reduction in motor plasticity.

The mechanism whereby premotor stimulation modulates motor plasticity is unknown. One possibility is that the premotor area modulates motor plasticity though heterosynaptic metaplasticity, a mechanism in which the history of synaptic activity not only alters subsequent synaptic plasticity in activated synapses but also in neighboring non-activated synapses [37], [38]. cTBS600 over PMd may lead to a form of heterosynaptic metaplasticity that changes the threshold for, or degree of response to cTBS300 over M1. The lack of such heterosynatic metaplasticity in our patients may coincide with the impaired homeostatic plasticity within M1 of WC patients [39]. It may be surprising to see the effect on cTBS is still present after 2 hr of premotor stimulation. However, the effect of metaplasticity is commonly seen after a protocol producing no plasticity effect on its own [40], [41] or after the end of detectable plasticity [42].

### Clinical Effects of Premotor Stimulation

Although this study was not designed as a clinical trial we did note that patients in the real group reported a more significant subjective improvement than those in the sham group, although this was not observed using objective measures of writing. This discrepancy implies either that the clinical improvement observed in the present study is contaminated by a placebo or learning effect or that the writing scores were insufficiently sensitive and the patient number is too small to detect relevant clinical changes.

Given the cumulative physiological effects of daily stimulation, it is possible that further sessions of premotor stimulation could further enhance clinical effects and distinguish sham and real groups. Similar delayed therapeutic benefit is found with DBS. DBS usually improves dystonia after weeks to months of continuous stimulation, whereas its physiological effect on synaptic plasticity occurs earlier [35].

In the sham group, we used the flip side of coil at a reduced intensity to deliver TBS. It feels and looks very similar to the real stimulation as compared to that tilts the coil away from the scalp or uses a sham coil with sound mimicking. Although the reduced intensity (from 80% to 60% AMT) may produce slightly different scalp sensation, subjects can barely distinguish between them when sessions are several days apart from each other. As a result, the lack of effect on all the physiological investigations even after five days of sham stimulation suggests that stimulating with a flip side coil is a good option for physiological investigations. However, we cannot fully rule out that the clinical improvement in the sham group was because of the very low stimulus intensity produced by the flip side coil.

## Conclusion

The present data show that multisession stimulation may not always prolong or enhance the effect of a single session. Indeed, in the present case, the effect on SICI observed after a single session was no different after 5 daily sessions. In contrast, multisession stimulation modulated the motor networks more extensively than a single session by restoring PMd-M1 interaction and reducing M1 plasticity. Thus testing with just a single session of rTMS may not give true insight into long-term effects of multisession stimulation. Moreover, the findings support previous arguments that clinical benefits of real stimulation could be further separated from those of sham by increasing the number of sessions, and provides a clearer rationale for using multisession brain stimulation to treat not only dystonia but also other diseases.

Finally, the present study provides additional physiological evidence that the premotor cortex is a potential target site for therapeutic intervention in the disordered motor networks of dystonia. It also highlights the potential importance of network-wide changes following intervention at a single site. Not only did we see changes in PMd-M1 connectivity but also in the response to plasticity protocols in M1. Given their potential benefits, remote effects from one area to another area deserve further study and may be relevant for treatment of related neurological disorders [43].
